# Birth-cohort HCV screening target in Thailand to expand and optimize the national HCV screening for public health policy

**DOI:** 10.1371/journal.pone.0202991

**Published:** 2018-08-23

**Authors:** Rujipat Wasitthankasem, Preeyaporn Vichaiwattana, Nipaporn Siripon, Nawarat Posuwan, Chompoonut Auphimai, Sirapa Klinfueng, Napha Thanetkongtong, Viboonsak Vuthitanachot, Supapith Saiyatha, Chaiwat Thongmai, Saowakon Sochoo, Panthip Sukthong, Kittiyod Poovorawan, Pisit Tangkijvanich, Yong Poovorawan

**Affiliations:** 1 Center of Excellence in Clinical Virology, Faculty of Medicine, Chulalongkorn University, Bangkok, Thailand; 2 Chumpare Hospital, Chum Phae, Khon Kaen, Thailand; 3 Phetchabun Provincial Public Health Office, Mueang Phetchabun, Phetchabun, Thailand; 4 Lomkao Crown Prince Hospital, Na-saeng, Lom Kao, Phetchabun, Thailand; 5 Lomsak Hospital, Lom Sak, Phetchabun, Thailand; 6 Department of Clinical Tropical Medicine, Faculty of Tropical Medicine, Mahidol University, Bangkok, Thailand; 7 Center of Excellence in Hepatitis and Liver Cancer, Department of Biochemistry, Faculty of Medicine, Chulalongkorn University, Bangkok, Thailand; Centers for Disease Control and Prevention, UNITED STATES

## Abstract

The World Health Organization aims to eliminate HCV infection worldwide by 2030. A targeted HCV screening policy is currently unavailable in Thailand, but a decrease in HCV infection has been observed in the country. However, a previous study showed that there was a higher HCV seroprevalence in adults aged between 30–64 years in the Phetchabun province (15.5%), as compared to the Khon Kaen province (3.6%). It was hypothesized that young adults had a lower rate of HCV seropositivity; this was determined by the age distribution of anti-HCV in Phetchabun and with the identification of high seroprevalence birth cohorts. In order to compare the provincial findings to the national level, anti-HCV birth cohorts were further analyzed in Khon Kaen (averaged-HCV prevalence) as well as the Thai data set that was derived from the previous literature. Thai individuals aged between 18–30 years residing in Phetchabun (n = 1453) were recruited, tested for the presence of anti-HCV antibodies and viral RNA and completed questionnaires that were designed to identify HCV exposure risks. Data was collected and compiled from previously published articles (n = 1667, age 30–64 years). The HCV seropositivity in Phetchabun by age group (18–64, at 5-year intervals) and the birth year were tabulated parallel to the Khon Kaen data set (n = 2233) in conjunction with data from the national survey 2014 (n = 5964) representing the Thai population. Factors such as age, male gender, agricultural work, blood transfusion, intravenous drug use and having a tattoo were associated with anti-HCV positivity in Phetchabun. HCV seroprevalence was less than 4.0% (ranging from 0.0–3.5%) from the age of 18–34 years. A dramatic increase of 15.1% was found in adults aged greater than or equal to 35 years, whereas, the age group in Khon Kaen and the national population with increasing prevalence of HCV were older (≥40). The HCV seropositivity cohort accumulated for those born between 1951–1982 accounted for 71.4–100.0% of all seropositive individuals. Subsequently, new cases occurred sporadically. This finding provides evidence that there is a disproportionately high HCV seroprevalence among people born before 1983 (or aged ≥35). This cohort should be targeted for priority screening as part of the national HCV screening policy. Incorporating this birth cohort with other risk factors could improve HCV diagnostic rates, resulting in overall improvements in parallel to those given by novel antiviral treatment.

## Introduction

Direct acting antiviral (DAA) treatments, the new curative medicines, have improved the viral therapeutic response and disease prognosis for chronic hepatitis C patients [1]. More affordable DAA treatments have increased the access to treatment among chronic hepatitis C patients, leading to a decrease in hepatitis C virus (HCV) transmission sources and HCV disease burden. Consequently, the World Health Organization (WHO) has set a goal for viral hepatitis to be eliminated worldwide by 2030 [2]. The aim of this strategy is to reduce the amount of new HCV infections and deaths by increasing the percentage of infected individuals diagnosed to 90%, with 80% of those being treated. Thailand has obtained voluntary licensing for drugs (such as sofosbuvir) that can provide effective treatment, which could potentially facillitate the elimination of hepatitis C. Nonetheless, for low- and middle-income countries (LMICs), obtaining a good diagnostic rate is difficult owing to several obstacles, including a lack of subpopulation targeting priority for HCV screening, and poor healthcare management of treatment for infected patients, which includes limited epidemiological information to aid the development of HCV elimination proactive strategies.

Insufficient HCV epidemiological data currently precludes viral management in Thailand due to the unavailablity of a reliable HCV screening policy, meaning there is a low diagnostic rate (~20%) that may be related to limited information on the HCV-related disease burden in the country [3]. With reference to the previous epidemiological research, Thailand has a relatively low HCV seroprevalence (0.9%) which seems to increase in the senior population [4]. However, a study in Phetchabun province was conducted on adults aged between 30–64 years which indicated a very high HCV infection rate with an overall seroprevalence of 15.5% and a viremic rate of 12.2% [5]. Seroprevalence in adults aged between 30–39 years (11.5%) was seven times higher than in the neighboring province, Khon Kaen, and a high proportion of advanced liver disease (52.2–62.5%) was found in chronic HCV carriers in these two areas [6]. These burdens may reflect past HCV infections (that occurred before implementation of the national HCV screening program in 1992) [7].

In order to elevate the diagnostic rate at the national level, a HCV screening policy developed from epidemiological data, including risk factors and target population, is required. Therefore, this study aimed to determine the provincial and national age distribution of HCV seropositivity, identifying the birth cohorts with high-seroprevalence as targets for priority screening. With regards to the the HCV prevalence magnitude in Phetchabun, we hypothesized that HCV seroprevalence might be much lower in young adults than in older adults. Furthermore, HCV seropositivity and potential risk factors in an expanded age group (18–64 years) were investigated in Phetchabun in conjunction with HCV birth cohort determination and the adjacent averaged-HCV prevalence was further analyzed for Phetchabun and Khon Kaen, as well as for the national survey, to generalize the findings in this study [4, 5]. Birth cohort expansion targeted to HCV screening (based on this information) would increase the diagnostic rate and would, therefore, be beneficial in determining recommendations for national policy.

## Materials and methods

The methodology involved blood sample collection from young adults residing in Phetchabun province in 2017. This research is an extension of the project “Prevalence and Genotypes of Hepatitis C Virus in Phetchabun and Khon Kaen Provinces as a Model for Treatment” [5]. The protocol was approved by the Institutional Review Board, the Faculty of Medicine, Chulalongkorn University with the amendment for additional participants aged between 18–30 years (IRB no 258/58, approval date April 27, 2017). The research protocol and methodology are in compliance with the overall aforementioned research project. The study objective was explained to the subjects and written informed consent was obtained from all participants.

### Study population

This study involved analyzing the seroprevalence of HCV based on the current and previously obtained data (Table 1) [4, 5]. Previously obtained HCV seroprevalence epidemiological data for Khon Kaen (a neighboring province to Phetchabun) were used for comparison [5]. A national survey conducted in 2014 was used to represent the hepatitis burden throughout the country [4]. The research methodology including the sample recruitment and laboratory assays was consistent across all of the studies [4, 5].

**Table 1:** HCV epidemiological data and previously published research. Epidemiological and virological data from both Phetchabun and Khon Kaen were combined. The HCV seroprevalence age distribution was analyzed for both sets of data including the national survey of HCV seroprevalence in 2014 which served as a HCV burden representative in the country [4].

Province	Collected Year	Age Range (years)	Sample Number	M/F	Anti-HCV^a^ (%)	HCV RNA (%)	HBsAg^b^ (%)	Demographic Data	Historical Risk Factors	Citation
Thailand^c^	2014	0–71	5964	2530/3434	56 (0.9)	23 (41.1)	1 (1.8)	Yes	No	[4]
Khon Kaen^d^	2014	18–61	823	286/537	18 (2.2)	13 (72.2)	0 (0.0)	Yes	No	[4]
Khon Kaen	2015	30–64	1410	556/854	51 (3.6)	31 (60.8)	2 (3.9)	Yes	Yes	[5]
Phetchabun	2015	30–64	1667	774/893	259 (15.5)	203 (78.4)	13 (5.0)	Yes	Yes	[5]
Phetchabun	2017	18–30	1453	709/744	16 (1.1)	8 (50.0)	1 (6.3)	Yes	Yes	This study

^a^Anti-HCV testing was consistent with the studies using automated ARCHITECT anti-HCV assay (Abbott Diagnostics).

^b^HBsAg are shown in anti-HCV positive samples.

^c^Samples collection from seven provinces of Thailand; Phra Nakhon Si Ayutthaya, Lop Buri, Uttaradit, Phitsanulok, Khon Kaen and Narathiwat.

^d^Samples derived from [4].

### Sample collection

With reference to the overall amended project mentioned above, the research methodology including sampling criteria, study protocol and laboratory assays were consistent with those used in the previous literature [5]. The sample collection was performed by expanding the population age range used in the previous study (30–64 years) by sampling from the same districts (Lom Kao and Lom Sak) in Phetchabun province [5]. Individuals were randomly recruited (n = 1453) from volunteers between July and August 2017 (Table 1). Participants were proportionally weighted to the population of each sub-district. The inclusion criteria focused on the general Thai population residing in Phetchabun, aged between 18–30 years, who generally experience good health and had no signs of chronic disease, had unknown HCV status, had no clinical symptoms associated with immunodeficiency disorder or HIV infection and no history of immunosuppressive therapy or HCV treatment. This age range was examined in compliance with the eligibility criteria of the National Health Security Office (NHSO) of Thailand for Universal Health Coverage (UC) Program reimbursement covering HCV screening, treatment and monitoring [6].

Demographic data and other potential risk factors associated with HCV infection were analyzed from the results of the completed questionnaires. The HCV risk exposures were analyzed from the history of blood transfusion, non-intravenous illicit drug use, intravenous drug use (IVDU), surgical history, sharp needle/injection administered by licensed medical person or unlicensed non-medical person, tattooing, sharing razors, acupuncture treatment, accidental needle stick injury, sexual orientation, having an HCV-infected spouse and having a family history of hepatitis disease or other liver disease, as described previously [5].

### Laboratory assays

Sera samples were tested for anti-HCV antibodies using an automated chemiluminescent microparticle immunoassay (ARCHITECT anti-HCV assay, Abbott Diagnostics, Wiesbaden, Germany). Reactive samples were designated as positive and subsequently tested for co-infection with hepatitis B virus (HBV) and/or human immunodeficiency virus (HIV) using automated hepatitis B surface antigen (HBsAg) and HIV antigen/antibody (Ag/Ab) assays (ARCHITECT, Abbott Diagnostics, Wiesbaden, Germany). Viral RNA was extracted in all anti-HCV reactive samples and nested RT-PCR was performed to amplify the viral core region. As previously described, the first-round PCR primers were 954F and 410R. The second-round PCR primers were 953F and 951R [8]. Samples with a positive HCV core result were considered to be viral RNA positive or representing an active infection. The expected 405 base-pair amplicons were purified and subjected to nucleotide sequencing. HCV sequences using BLASTN were preliminarily assigned to genotypes [9].

### Data analysis

There were three main data sets: Phetchabun, Khon Kaen and the national survey (of which the age of interest was between 18–64 years) (Table 1). The Phetchabun data set consisted of integral information of the socio-demographic factors, HCV risk exposures and HCV seroprevalence obtained from the current study and from the previously reported 2015 data [5]. Similarly, the data from two HCV epidemiological cohorts for Khon Kaen province (collected in 2014 and 2015) were combined to give the Khon Kaen data set [4, 5]. In addition, the national survey results that were derived from the HCV national study findings published in 2014 were used as the general Thai population reference data [4].

HCV seroprevalences were stratified from 5-year intervals, except for individuals aged 18–19 years for which a 2-year interval was used. Birth and fertility rates were obtained from the World Bank Database [10, 11]. The birth year was simply calculated from the data collection year and the individual’s age.

### Statistical analysis

Factors associated with HCV seropositivity were evaluated solely from the Phetchabun data set due to incomplete information on HCV risk factors in the Khon Kaen data set collected in 2014. With regard to the statistical analysis, SPSS Statistics version 22 (IBM Corporation, Armonk, NY) was used. A Pearson chi-square or Fisher’s exact tests for categorical variables were used to make comparisons between the groups. Logistic regression was used to identify the effects of potential risk factors for HCV positivity in the univariate and multivariate analysis. Factors with a *p*-value less than 0.2 in the univariate analysis were further analyzed for independent effects in a multivariate logistic regression model, and a *p*-value of less than 0.05 was considered statistically significant. Odds ratios (ORs), 95% confidence intervals (CIs) for demographic factors and other risk factors were derived from the univariate and multivariate analyses.

## Results

### HCV seroprevalence and risk exposures in Phetchabun

The mean age of participants recruited in Phetchabun for this study and providing characteristics samples was 23.7 ± 3.7 years within the age range of 18–30 years, based on 709 males and 744 females (Table 2). The majority of participants possessed high school education and were temporary employees. Positive samples of anti-HCV (1.1%, 16/1453) were mainly found in older age groups. The positivity was solely associated with the age range (*p*-value = 0.021). Samples that tested positive for HCV antibodies were HBsAg positive at 6.3% (1/16) with no HIV Ag/Ab positivity, and HCV RNA positivity was found in 8 samples, the following HCV genotypes were found; 1a (1 sample), 1b (2 samples), 3a (4 samples) and 6f (1 sample).

**Table 2:** Phetchabun demographic and HCV seroprevalence of the samples (age between 18–30 years) recruited in 2017.

	Total	Anti-HCV Positive (%)^a^
**Mean Age (SD)**	23.7 (3.7)	25.8 (2.6)
**Age Range (years)**^b^		
18–19	263	0 (0.0)
20–24	550	4 (0.7)
25–29	551	9 (1.6)
30	89	3 (3.4)
TOTAL	1453	16 (1.1)
**Gender**		
Male	709	9 (1.3)
Female	744	7 (0.9)
**Education**		
≤Grade 1–6	158	3 (1.9)
Grade 7–9	361	5 (1.4)
Grade 10–12	513	6 (1.2)
University or higher	420	2 (0.5)
TOTAL	1452	16 (1.1)
**Occupation**		
Agriculture	375	6 (1.6)
Temporary employee	520	5 (1.0)
Non-agriculture	439	5 (1.1)
TOTAL	1334	16 (1.2)

^a^Percentage calculated according to each characteristic.

^b^Statistical significant association tested by Fisher’s exact test between group differences (*p*-value < 0.05).

**Fig 1:**
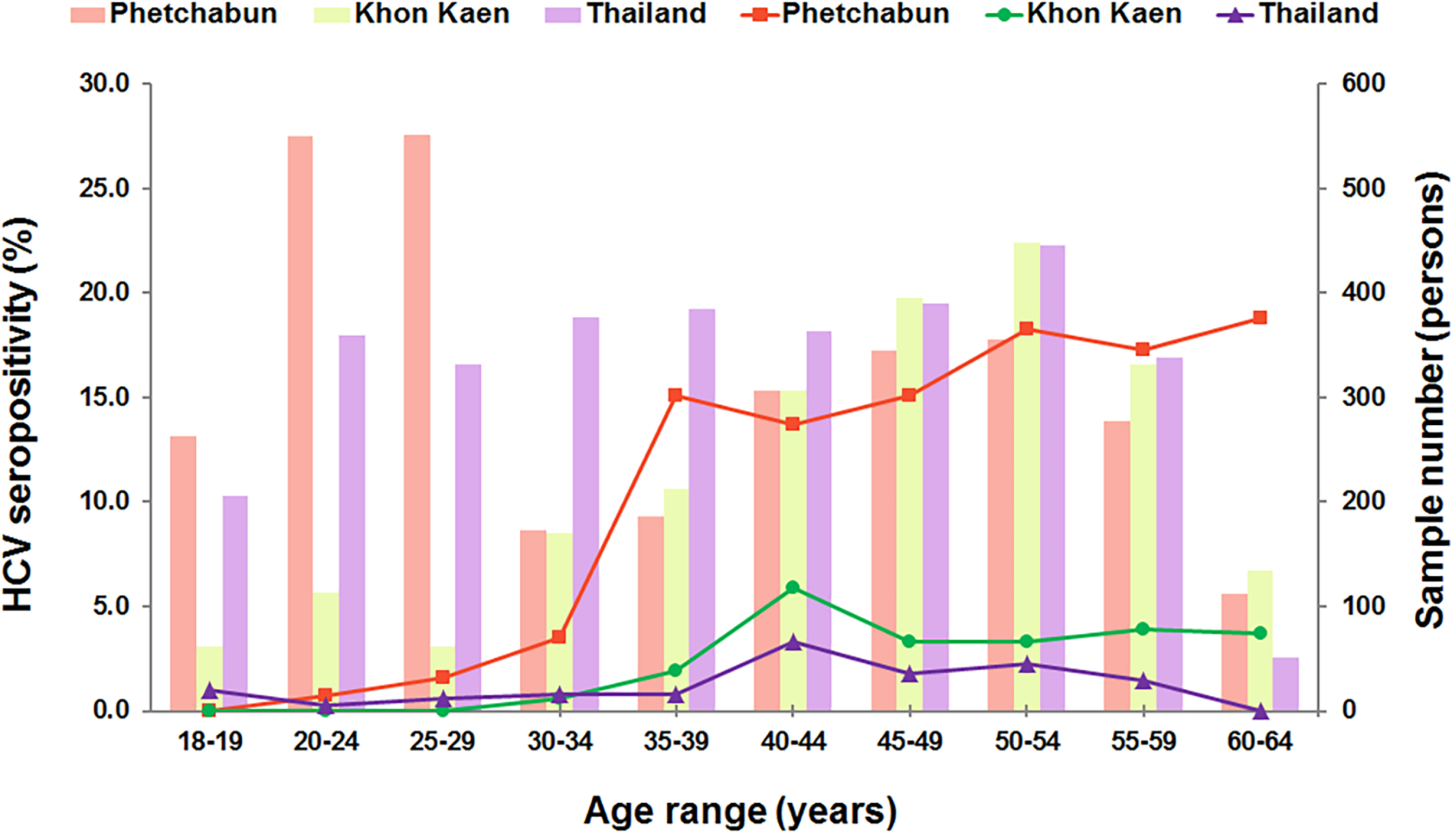
HCV seroprevalence in different age groups. HCV seroprevalence in individuals aged 18–64 years in Phetchabun (red color), Khon Kaen (green color) and Thailand (purple color). Data were categorized into 5-year or 2-year interval. The line and bar graphs show HCV seropositivity and individual sample numbers in each age group, respectively. Khon Kaen and the national survey data were derived from previous studies [4,5].

The current data (aged 18–30 years) and that of the previous cohort from individuals aged 30–64 years in Phetchabun province [5] were subsequently combined to evaluate the association between the risk factors and HCV seropositivity. Integral samples (n = 3120) for the Phetchabun data set (aged 18–64 years) were categorized into age groups using 5-year intervals, except for those individuals aged 18–19 years, for which a 2-year interval was used (Fig 1). The HCV seroprevalence was relatively low (0.0–3.5%) for individuals aged less than 35 years and increased sharply to 15.1% for people aged greater than or equal to 35 years. The seroprevalence tended to be fairly steady (and slightly increased) in the 50 years or more age group (18.3–18.8%).

Several risk exposures were found to be statistically significant including age, gender, education, occupation, and history of oral drug abuse and IVDU (Table 3) in the HCV seropositivity univariate analysis for the Phetchabun data set. Individuals who tested positive for HCV antibodies were more likely to have undergone medical treatment involving injection either by medical or non-medical staff, shared sharp objects or razor blades or had a tattoo.

**Table 3:** Univariate and multivariate analyses of potential risk factors associated with HCV seroprevalence in Phetchabun.

			Univariate analysis		Multivariate analysis	
Parameters	All	Anti-HCV (%)	OR (95% CI)	*p*-value	OR (95% CI)	*p*-value
**Age**_**10**_^a^	3120	275 (8.8)	2.1 (1.9, 2.3)	<0.001	1.9 (1.7, 2.3)	<0.001
**Gender (M:F)**	1483/1637	229 (15.4)/46 (2.8)	6.3 (4.6, 8.7)	<0.001	4.7 (3.2, 6.9)	<0.001
**Education**	3091					
≤ Grade 1–6	1103	172 (15.6)	5.3 (3.8, 7.3)	<0.001	1.3 (0.8, 2.0)	0.282
Grade 7–9	631	52 (8.2)	2.6 (1.7, 3.9)	<0.001	1.6 (1.0, 2.6)	0.051
≥ Grade 10	1357	46 (3.4)	1.0 (-, -)		1.0 (-, -)	
**Occupation**	2961					
Agriculture	1486	210 (14.1)	6.7 (4.0, 11.3)	<0.001	2.9 (1.6, 5.4)	0.001
Temporary employee^b^	806	41 (5.1)	2.2 (1.2, 3.9)	0.009	1.7 (0.8, 43.3)	0.161
Non-agriculture^c^	669	16 (2.4)	1.0 (-, -)		1.0 (-, -)	
**Blood transfusion**	3044		1.4 (0.9, 2.2)	0.137	1.8 (1.1, 3.2)	0.041
No	2850	248 (8.7)				
Yes	194	23 (11.9)				
**Oral drug**	3071		5.0 (3.4, 7.3)	<0.001	1.2 (0.7, 2.2)	0.548
No	2923	225 (7.7)				
Yes	147	43 (29.3)				
**Intravenous drug use**	3063		20.0 (11.7, 34.2)	<0.001	9.1 (4.3, 19.1)	<0.001
No	3002	229 (7.6)				
Yes	61	38 (62.3)				
**History of surgery**	3077		1.0 (0.7, 1.3)	0.768	-	-
No	2218	196 (8.8)				
Yes	859	73 (8.5)				
**Injection by medical staff**	2996		1.8 (1.4, 2.3)	<0.001	1.0 (0.8, 1.5)	0.773
No	1854	132 (7.1)				
Yes	1142	137 (12.0)				
**Injection by non-medical staff**	2999		2.2 (1.6, 3.1)	<0.001	1.2 (0.8, 1.9)	0.311
No	2659	212 (8.0)				
Yes	340	55 (16.2)				
**Acupuncture**	3032		1.5 (0.7, 3.4)	0.299	-	-
No	2977	259 (8.7)				
Yes	55	7 (12.7)				
**Needle stick**	3076		1.1 (0.5, 2.2)	0.817	-	-
No	2981	262 (8.8)				
Yes	95	9 (9.5)				
**Sharing sharp object or razor blade**	3076		2.1 (1.5, 2.9)	<0.001	1.0 (0.6,1.6)	0.974
No	2764	223 (8.1)				
Yes	312	48 (15.4)				
**Tattooing**	3075		3.2 (2.5, 4.2)	<0.001	1.7 (1.2, 2.4)	0.001
No	2464	159 (6.5)				
Yes	611	111 (18.2)				
**HCV-infected spouse**	3054		0.9 (0.5, 1.9)	0.827	-	-
No	2945	261 (8.9)				
Yes	109	9 (8.3)				
**Hepatitis within family**	3055		0.8 (0.5, 1.1)	0.178	0.9 (0.5, 1.4)	0.512
No	2614	235 (9.0)				
Yes	441	31 (7.0)				
**Homosexual**	3021		0.9 (0.6, 1.5)	0.720	-	-
No	2757	248 (9.0)				
Yes	264	22 (8.3)				

^a^Age stratified by a 10-year interval.

^b^Temporary employee: mixed between agriculturist and non-agriculturist.

^c^Non-agriculture occupation consisted of business owner, government employee, state enterprise employee, medical/hospital worker, monkhood and others.

Twelve variables (*p*-value <0.2) were further included in the multivariate analysis to identify the independent effects of the risk factors. In Phetchabun, HCV seropositivity was independently associated with increasing age (in 10–year intervals, adjusted OR 1.9, 95% CI 1.7–2.3), male gender (adjusted OR 4.7, 95% CI 3.2–6.9), occupation related to agricultural employment (adjusted OR 2.9, 95% CI 1.6–5.4), blood transfusion (adjusted OR 1.8, 95% CI 1.1–3.2), IVDU (adjusted OR 9.1, 95% CI 4.3–19.1) and having had a tattoo (adjusted OR 1.7, 95% CI 1.2–2.4) configured after adjustment for education, history of oral drug use, therapeutic injection by medical or non-medical staff, sharing sharp objects and having a family history of hepatitis disease.

### The age distribution and HCV seroprevalence in Phetchabun, Khon Kaen and the national study

Fig 1 shows the age-dependent HCV seroprevalence in Phetchabun, Khon Kaen and the national survey. In comparison to the other two data sets, higher HCV seroprevalence was observed in almost all age groups in Phetchabun. Low HCV seropositivity was observed in young adults, but this dramatically increased in people aged 35 years old or more with a peak prevalence (18.8%) at 60–64 years. Similar to the results found for Phetchabun, in both Khon Kaen and the national survey, the rate was low in younger generation but increased in the 40 years or older age group. The peak prevalence was 40–44 years of age (5.9% in Khon Kaen and 3.3% in the national survey) and slightly declined and became steady after this age. Therefore, a similar age distribution for HCV seroprevalence was found in Khon Kaen and the general population, even though Khon Kaen had a moderately higher anti-HCV positive rate.

### The birth cohort and HCV seroprevalence in Phetchabun, Khon Kaen and the national study

**Fig 2:**
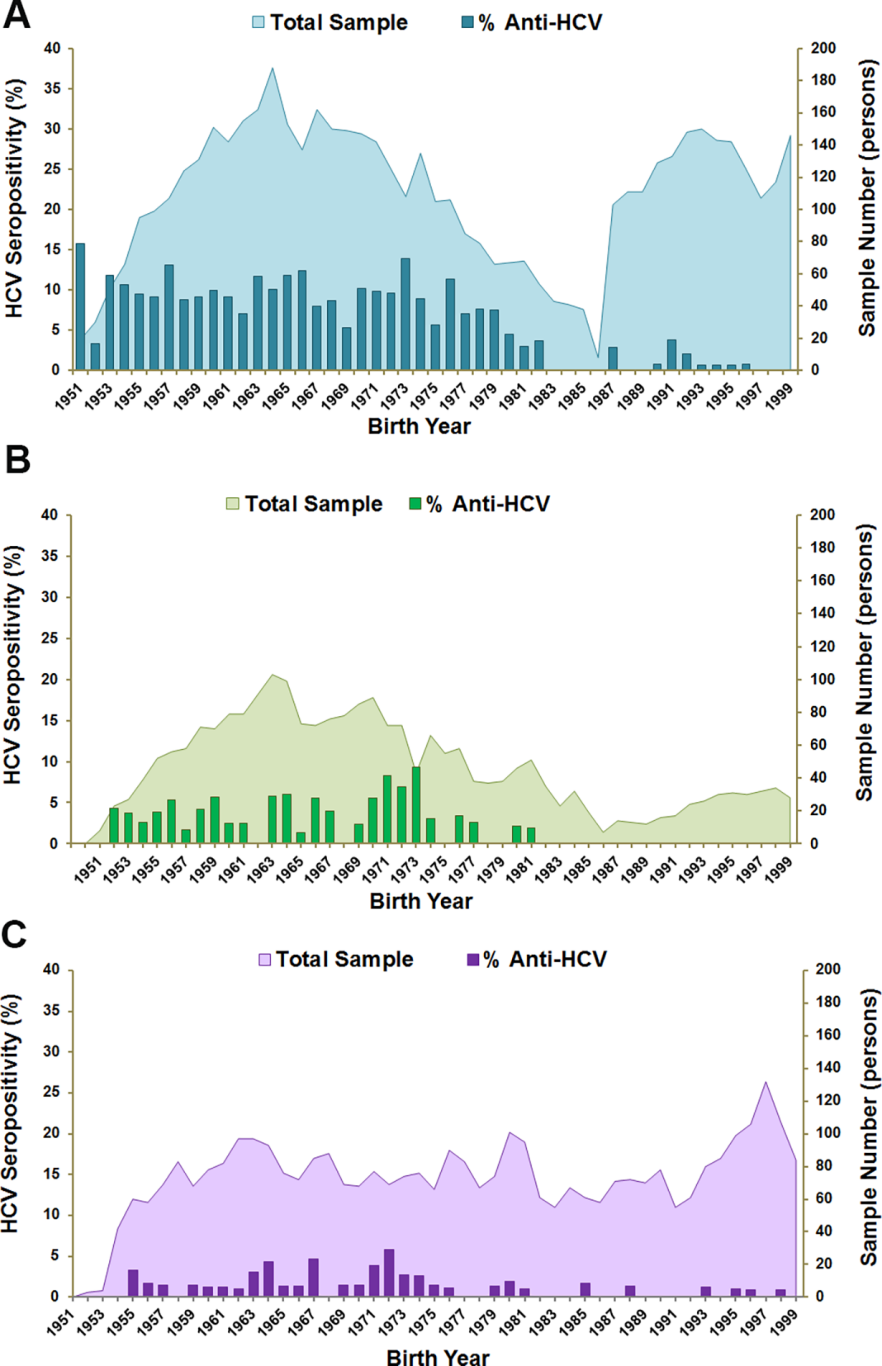
HCV seroprevalence in different birth cohorts. The bar charts show HCV seroprevalence in birth cohorts born in 1951–1999 in (A) Phetchabun, (B) Khon Kaen Province and (C) the national survey. The area behind the bar chart represents the sample number in each birth year.

To identify the birth cohort with the highest HCV seroprevalence, the seropositivity was analyzed according to each birth year between the years 1951–1999 (Fig 2). In Phetchabun, HCV seroprevalence was steadily high in the 1951–1982 birth cohort, with sporadic cases afterwards (Fig 2A). Khon Kaen, the adjoining province, had a lower degree of HCV prevalence as compared to Phetchabun. The antibodies positivity was accumulated in people born in 1951–1981 and was undetected in individuals born after 1981 (Fig 2B). Likewise, the national survey showed a disproportionate rate of positive anti-HCV results for people born in 1955–1981 and revealed sporadic positive HCV antibodies results after 1981 (Fig 2C).

**Fig 3:**
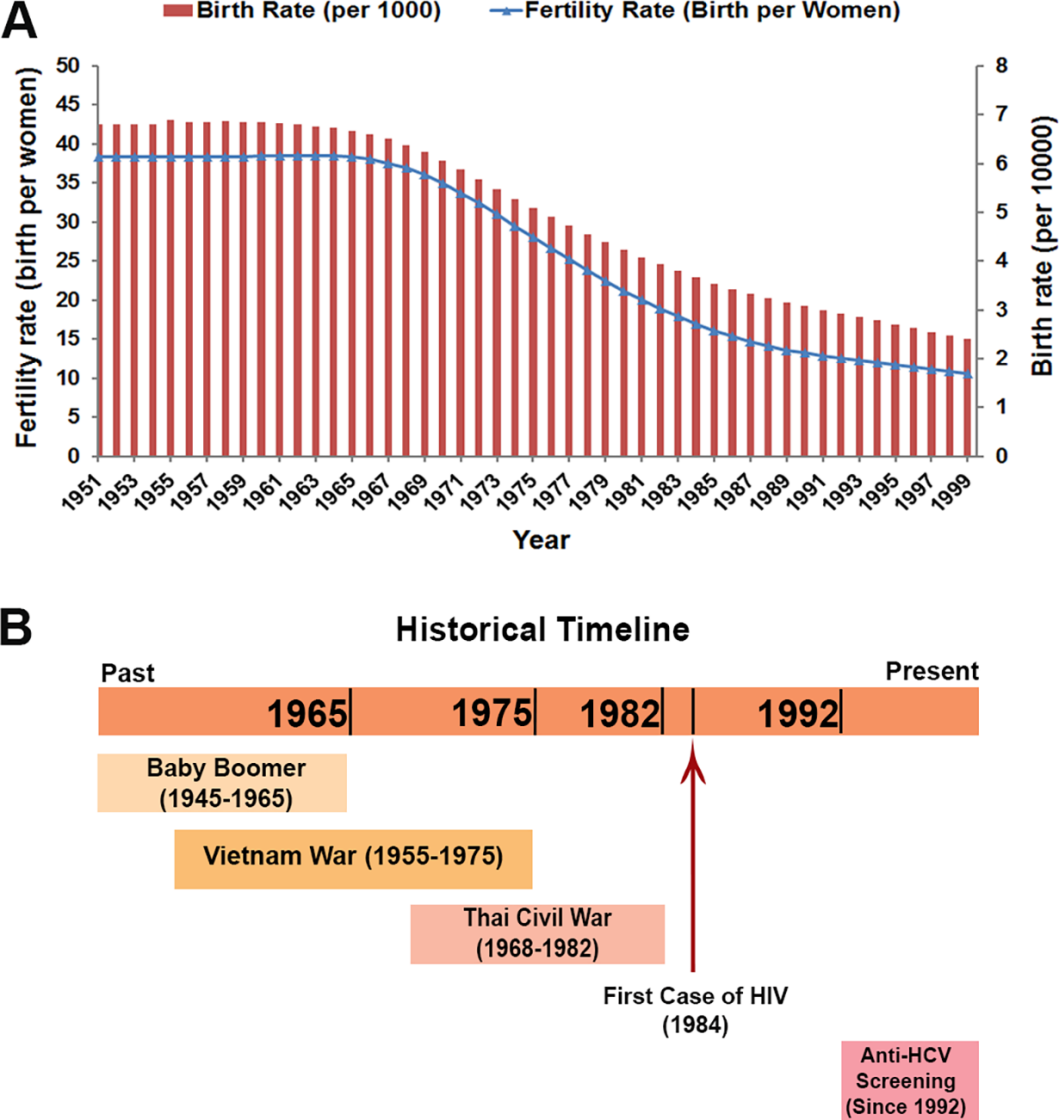
Fertility and birth rates of Thai population born in 1951–1999 and HCV related historical timeline transmission in Thailand. (A) The bar and line graph respectively show the birth rate per 1000 population and birth fertility rate per woman during 1951–1999 in Thailand. These data are derived from the World Bank database [10,11]. (B) The bar charts show historical timeline and socioeconomic events that may be related to HCV prevalence and transmission in Thailand.

The fertility and birth rates in Thailand during 1951–1999 show that the baby boomer period started before 1951 and went on until 1965 when the fertility rate began to decrease gradually (Fig 3A). Table 4 shows the HCV screening target according to several birth cohorts: baby boomer, Vietnam War period and people born before 1983. Using the baby boomer cohort (born in 1945–1965) as a target screening group could cover the low anti-HCV percentage (28.6–47.8%) from all positive individuals, as compared to the other birth cohorts. On the other hand, the birth cohort of those born before 1983 showed the highest coverage, 71.4–100% in all data sets. Moreover, people aged 35 years or older were significantly associated with increased ORs to anti-HCV positivity (Phetchabun, OR 15.4, 95% CI 9.6–24.7, *p*<0.001; Khon Kaen, OR 15.6, 95% CI 2.2–112.7, *p* = 0.006 and the national survey, OR 3.0, 95% CI 1.4–6.5, *p* = 0.005). Hence, the birth cohort born before 1983 seems to be a good target for HCV screening priority for public health policy.

**Table 4:** HCV seroprevalence from individuals born in different birth cohort periods (the baby boomer and Vietnam War periods) including target birth cohort from Phetchabun, Khon Kaen data and the national survey.

	Anti-HCV (positive^a^/total^b^)	Coverage (%)
**Thailand**		
Baby boomer^c^ (1945–1965)	16/56	28.6
Vietnam War^d^ (1955–1975)	35/56	62.5
Born before 1983^e^	40/56	71.4
**Khon Kaen**		
Baby boomer^c^ (1945–1965)	33/69	47.8
Vietnam War^d^ (1955–1975)	61/69	88.4
Born before 1983^e^	69/69	100
**Phetchabun**		
Baby boomer^c^ (1945–1965)	137/275	49.8
Vietnam War^d^ (1955–1975)	214/275	77.8
Born before 1983^e^	259/275	94.2

^a^Anti-HCV positive number by applying each birth cohort target.

^b^Total positive samples obtained in the national survey (total positive number = 56), Khon Kaen (total positive number = 69) and Phetchabun data set (total positive number = 275).

^c^Baby boomer cohort born during 1945–1965.

^d^Vietnam War cohort born during 1955–1975.

^e^Proposed birth cohort born before 1983.

## Discussion

The current prevalence of HCV may be as a result of past infection earlier in life. Previous studies indicated that HCV seroprevalence was high in adults age between 30–64 years and was associated with several risk factors and exposures including male gender, having a history of IVDU and having a tattoo [5, 12] whereas the level of educational and an agricultural related occupation were found to be additional risks to HCV infection in the high prevalence area of Phetchabun province when compared to Khon Kaen, where there was average prevalence. In order to investigate the HCV status in a younger age group, we examined the seroprevalence in adults aged 18–30 years and integrated the data with those from the previous study resulting in an expanded age range covering from 18–64 years, including the analysis of the overall risk factors and current HCV seroprevalence in Phetchabun [5]. As expected, the results showed very low seroprevalence in the younger generation, as well as an association between risk factors (age, male gender, agricultural occupation, blood transfusion, history to IVDU and tattoo exposures) and anti-HCV positivity. The low HCV prevalence in young adults indicated that Thai public health has improved since the introduction of HCV mandatary screening and universal precautions related to the prevention of the transmission of bloodborne diseases, even in a severely endemic area, which is consistent with the previous findings [4, 5, 7].

Socioeconomic factors and other factors have been reported to increase the prevalence of HCV infection. For instance, the national anti-schistosomal injection campaign in Egypt [13], high IVDU, and blood transfusion rates during World War II in Japan and the Civil War in Spain have all been correlated with high HCV transmission rates [14]. In Thailand, HCV prevalence seems to have coincided with poor precautions for transmission of bloodborne diseases during blood transfusions and sharing of needles by people who were born during the baby boomer period (1945–1965) (Fig 3A) [15]. Likewise, the IVDU that was widespread during the Vietnam War (1955–1975) may also have been a factor [16]. In addition, the sampled study area of Phetchabun province was an area in which there was a Thai Civil War between the civilians and the government during 1968–1982 [17, 18]. Several camps were set up, and many schools and hospitals had inadequate equipment, therefore these factors could have promoted HCV transmission during this period (Fig 3B). The war ended in 1982, dramatically decreasing the prevalence as was consistently observed in this study (Figs 2 and 3B).

In 2016, the WHO developed global guidelines for targeted HCV testing of populations [19]. The recommendations of the guidelines included focused testing in the most affected populations that have high-risks of exposure or behaviors for HCV infection (e.g. people who inject drugs, incarcerated people, men who have sex with men, sex workers, those who are infected with HIV, or people with clinical suspicion of chronic hepatitis). In countries that have an overall prevalence of greater than 2–5%, general population testing should be carried out in addition to birth cohort testing in specifically identified birth cohorts or older people known to have a high risk of HCV infection and morbidity within an overall lower general prevalence.

A reliable screening policy with information on the birth cohort population (with higher risk to HCV infection) for which a screening target should be applied is still unavailable in Thailand. The majority of new HCV cases identified were due to clinical suspicion and risk-based assessment during hospitalization or blood donation. In addition to the risk based HCV testing approach, evidence from many countries shows an increased diagnostic rate when some birth cohort population components (*i*.*e*. the baby boomer period) are incorporated into HCV testing recommendations [20–25]. Our results showed the evidence of an unevenly high prevalence of anti-HCV in the population born before 1983 (1982 cut-off year) or those aged 35 years or more, and were derived from low-, average- and high prevalence settings. The majority of HCV seropositivity accounted for 71.4–100% in people born in this cohort (Table 4). The age cohort found to have increasing HCV antibody prevalence was between 35 (in the high prevalent area) and 40 years (in average prevalence and the general population). Therefore, to generalize these finding, we recommend HCV testing in people born before 1983 and covering individuals aged 35 years or more as a target screening cohort.

One-time testing in the baby boomer birth cohort effectively identified HCV infection in US and European countries. This approach had a significant impact on the HCV diagnostic rate and cost-effectiveness of diagnoses [21–25]. In 2012, the US Centers for Disease Control and Prevention (CDC) released new recommendations for one-time HCV screening in baby boomer cohorts, independent of their risk exposure [26]. By following this strategic implementation, the screening rate in the US increased by 49%, followed by an 86% increase in identification of undiagnosed cases among the population born in 1945–1965 [27, 28]. Moreover, in France, enhanced HCV screening in this cohort, together with corresponding curative DAA treatment, was cost-effective and may reduce the future HCV burden [24].

One-time HCV testing in the general population has been recommended in countries with a high prevalence of above 2–5%. A literature review showed that the prevalence of HCV infection leading to increasing numbers of case finding was a key to cost-effectiveness in different screening approaches (with links to treatment) [20]. The study suggested that screening in high prevalence settings such as people who inject drugs and particular birth cohorts would be likely to prove cost-effective, including routine general population testing in the LMICs with more than or equal to 2% anti-HCV prevalence. In addition, a recent simulation analysis in the US suggested that routine HCV testing in all adults (aged ≥18 years) would be cost-effective owing to the increasing incidence of new HCV infections via IVDU in young adults [29]. In the case of the population of Thailand, the overall HCV seroprevalence was approximately 0.9%, with 0.4% seropositivity in people born outside of those birth cohort areas (1982 cut-off year or age <35 at present) [4], therefore, epidemiological studies and cost-effectiveness of targetted HCV screening outside of these birth cohorts should be further investigated. Disregarding the general population testing, birth cohort HCV testing together with focused testing in high-risk groups (agricultural workers, blood transfusion, IVDU and tattoo exposure) are suggested as targeting priorities and appropriate to establish a national screening policy suitable for the current endemic context of the country.

The standard HCV diagnostic algorithm consists of two steps; serological HCV antibodies screening and confirmatory active infection screening using nucleic acid testing. Viral genotyping is required before initiating a conventional IFN-based treatment. Owing to costly assays and infrastructure requirements, these complex approaches limit access to treatment and care system, particularly in LMICs. As novel HCV therapeutic costs decline, expensive assays may not be necessary. Thus, a simplified screening strategy would be necessary to increase the HCV diagnostic rate, such as introducing rapid diagnostic tests to screen for anti-HCV in resource-constrained settings. Rapid diagnostic tests are easily accessed using finger-stick whole blood or oral fluid specimens [30]. Instead of nucleic acid testing, several studies have suggested that supplementary testing with the HCV core antigen (HCVcAg) was cost-effective and could potentially identify an active HCV infection [31–33]. These approaches could give promising results in the near future.

The current study had several limitations. Primarily, there was a small number of anti-HCV-positive samples in the national study which was used to represent the general population in Thailand [4]. Secondly, the birth cohort identified for targeted screening in this study was identified based on three data sets due to the lack of a large surveillance study. Therefore, further studies should be conducted in several research areas based on larger samples randomly selected from every province to evaluate the effectiveness of the risk-based plus birth cohort HCV testing strategy to optimize the screening approach algorithm in the context of national level implementation. The cost-effectiveness of these approaches should also be considered.

This study shows a disproportionately high HCV seroprevalence in the population of people born before 1983. This population is at risk of developing severe liver diseases, especially in endemic areas. To achieve the WHO goal of eliminating HCV infection by 2030, the Thai Ministry of Public Health should primarily set targets for HCV populations with high prevalence (e.g. high-risk groups and birth cohorts), implement proactive priority screening in the coming years and establish national screening recommendation guidelines. We propose HCV screening based on this birth cohort (those born before 1983), in addition to targeting groups with other risk exposures, should be a national policy. This strategy would help to increase the diagnosed population and coupled with DAA treatment; it could enhance HCV elimination in the near future.
